# Language in schizophrenia: relation with diagnosis, symptomatology and white matter tracts

**DOI:** 10.1038/s41537-020-0099-3

**Published:** 2020-04-20

**Authors:** J. N. de Boer, M. van Hoogdalem, R. C. W. Mandl, J. Brummelman, A. E. Voppel, M. J. H. Begemann, E. van Dellen, F. N. K. Wijnen, I. E. C. Sommer

**Affiliations:** 1 Department of Psychiatry, University Medical Center Utrecht, Utrecht University & Brain Center Rudolf Magnus, Utrecht, The Netherlands; 2 Department of Neuroscience and Department of Psychiatry, University Medical Center Groningen, University of Groningen, Groningen, The Netherlands; 3 Department of Intensive Care Medicine, University Medical Center Utrecht, Utrecht, The Netherlands; 4 Department of Psychiatry, OLVG, Amsterdam, The Netherlands; 5 Department of Psychiatry, Melbourne Neuropsychiatry Centre, University of Melbourne and Melbourne Health, Melbourne, VIC Australia; 6 Utrecht Institute of Linguistics OTS, Utrecht University, Utrecht, The Netherlands

**Keywords:** Human behaviour, Neural circuits, Schizophrenia

## Abstract

Language deviations are a core symptom of schizophrenia. With the advances in computational linguistics, language can be easily assessed in exact and reproducible measures. This study investigated how language characteristics relate to schizophrenia diagnosis, symptom, severity and integrity of the white matter language tracts in patients with schizophrenia and healthy controls. Spontaneous speech was recorded and diffusion tensor imaging was performed in 26 schizophrenia patients and 22 controls. We were able to classify both groups with a sensitivity of 89% and a specificity of 82%, based on mean length of utterance and clauses per utterance. Language disturbances were associated with negative symptom severity. Computational language measures predicted language tract integrity in patients (adjusted *R*^2^ = 0.467) and controls (adjusted *R*^2^ = 0.483). Quantitative language analyses have both clinical and biological validity, offer a simple, helpful marker of both severity and underlying pathology, and provide a promising tool for schizophrenia research and clinical practice.

## Introduction

Language disturbances are a core symptom of schizophrenia. Since the first descriptions of schizophrenia as a mental disorder, language disturbances have been referred to as formal thought disorder (FTD)^1^. Kraepelin identified a subgroup of patients with severe confusion of speech, a symptom he described as “schizophasia”, characterized by “an unusually striking disorder of expression in speech, with relatively little impairment of the remaining psychic activities”^1^. Indeed, there is abundant evidence that language disorder is a key symptom of schizophrenia^2–6^. We aim to study the relation between language disturbances and schizophrenia, its symptomatology, and the underlying neurobiology.

Language disturbances in schizophrenia are multidimensional. Positive language symptoms include idiosyncratic semantic associations, neologisms and word approximation^3,4,7^. Negative language symptoms are poverty of speech (ranging from less frequent and slower to complete absence), and reduced grammatical complexity^5,8,9^.

Until recently, studies have used subjective observation-based instruments to investigate language disturbances in schizophrenia^10^. Although these rating scales have clinical utility, they do not support the assessment of subtle phenomena/deviations with respect to language form (i.e., grammar as well as sound structure). With the availability of automatic speech recognition and computational linguistic tools, which provide easy and fast quantitative analyses of phonetics, syntax and semantics^11–14^, language can easily be assessed in exact and reproducible measures. However, studies applying quantitative analyses of language in the field of schizophrenia have so far been scarce^12,14^.

To date, research on the neurobiological underpinnings of language disturbances in schizophrenia is limited. Cumulative evidence suggests that symptoms associated with schizophrenia may be the result of disordered brain connectivity^15–17^. White matter structural organization can be studied in vivo by means of diffusion tensor imaging (DTI). A recent meta-analysis from the ENIGMA Working Group on schizophrenia DTI suggests that white matter alternations in schizophrenia are widespread^18^; disturbances were found in almost all regions analyzed. Furthermore, FTD in schizophrenia has been associated with both structural and functional aberrations in the language network^19^. Moreover, language connectedness indicated by speech graph analysis was related to functional as well as structural brain markers (cortical folding patterns) in both schizophrenia and bipolar disorder^20^. However, to date there are no studies investigating white matter microstructure of the language pathways and their association to language disturbances specifically.

**Fig. 1:**
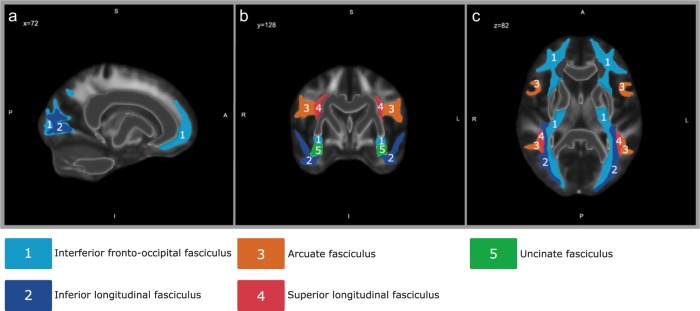
Regions of interest. Depiction of the five regions of interest (ROIs) of the The Johns Hopkins University (JHU) DTI-based white matter atlases^82,83^, that were analyzed in the present study in the sagittal plane (**a**), the coronal plane (**b**) and the axial plane (**c**).

Dual-stream models associate distinct functions with left and right hemisphere language networks. The ventral stream, connecting the temporal cortex (including Wernicke’s area) with Broca’s area, supports sound-to-meaning mapping, whereas the dorsal pathway, from the posterior temporal lobe to the premotor cortex as well as the pars opercularis of Broca’s area, is taken to support auditory–motor integration^21^. In the current study, we used DTI to study the microstructure of the language pathways. Regions of interest (ROIs) were selected using reviews and meta-analyses on the white matter language network^22–24^. Both the dorsal stream (superior longitudinal fasciculus (SLF) and arcuate fasciculus (AF)) as well as the ventral stream (inferior longitudinal fasciculus (ILF), inferior fronto-occipital fasciculus (IFOF) and uncinate fasciculus (UF)) were included (Fig. 1). Fractional anisotropy (FA) and mean diffusivity (MD) were used as an index for white matter integrity^25^.

We evaluated whether quantitative analyses of spoken language can be used to (1) classify subjects as schizophrenia patients or controls, thereby assessing its potential to aid in diagnosis. In addition, we evaluated how language characteristics relate to (2) schizophrenia symptoms and (3) structural integrity of the language pathways.

## Results

### Demographics

Demographic characteristics are listed in Table 1. Healthy controls and patients with schizophrenia on average did not differ in age, gender distribution or educational level. To assess the effect of antipsychotic treatment on our analyses, correlation analyses were performed. No significant relations were found between chlorpromazine equivalent dosage and Positive And Negative Syndrome Scale (PANSS)^26^ sub scores (all *p* > 0.400), language measures (all *p* > 0.100) or language tracts (Supplementary Tables 1, 2).

**Table 1:** Demographic characteristics of the study sample.

	Schizophrenia patients	Healthy controls	Test statistics
*N*	26	22	
Age, mean (SD)	26.7 (5.43)	24.3 (4.40)	*F* = 1.436, *p* = 0.237
Gender, *n* (%) male	20 (76.9)	19 (86.4)	χ^2^ = 0.697, *p* = 0.478
Years of education			
Participant, median (IQR)	14 (11.5–16.5)	15 (12.5–17.5)	MW: *p* = 0.052
Parental, median (IQR)	14.5 (12.8–16.3)	14.5 (12.4–16.6)	MW: *p* = 0.982
Illness duration in years, mean (SD)	2.1 (1.50)		
Diagnosis, *n* (%)			
Psychosis NOS	13 (50.0)		
Schizoaffective disorder	2 (7.7)		
Schizophrenia	8 (30.8)		
Schizophreniform disorder	3 (11.5)		
PANSS, mean (SD)			
Positive	10.8 (3.94)		
Negative	14.9 (5.47)		
General	26.7 (6.28)		
Total	52.4 (11.42)		

*n* sample size, *SD* standard deviation, *Md* median, *IQR* inter quartile range, *MW* Mann–Whitney *U*, *PANSS* positive and negative syndrome scale, *NOS* not otherwise specified.

### Diagnostic categories

The MANCOVA comparing both groups including age as a covariate, revealed a significant main effect of group status on language characteristics (*F*_(11,35)_ = 2.565, Pillai’s trace = 0.446, *p* = 0.017) (Tables 2, 3). No main effect was found for age. Post hoc testing revealed that patients articulated more slowly, spoke during a smaller proportion of the interview, produced shorter utterances, had a higher type-token ratio (TTR, i.e., a measure for lexical diversity) and used fewer clauses per utterance than the healthy controls (all *p*’s < 0.050, Table 2).

**Table 2:** Description of language variables.

Variable	Definition/calculation	Measures
Articulation rate	Syllables/phonation time	(Motor) speed in speech production
Average pause duration	Total time the participant was pausing in seconds/number of pauses	Pauses often reflect formulating or planning language and might therefore reflect processing speed
Average turn duration	Average duration of a speaking turn in seconds	Average length of an answer, before another question is necessary
Percentage of time speaking	Time participant speaking/time interviewer speaking × 100	Might reflect spontaneity in speech or willingness to speak
Mean length of utterance (MLU)	Mean length of utterance in morphemes	Sentence complexity. Greater length indicates more complex sentences
Type-token ratio (TTR)	# types/# tokens	Lexical diversity. Types: the number of different words used in the sample. Tokens: all words in the sample. This number goes from 0.001 to 1.0. Low values indicate a lot of repetition, high values means each word in the sample was different. High TTR indicates fewer syntactical structures
Clauses per utterance	Average number of clauses per utterances	Grammatical complexity. More clauses per utterance indicate more syntactical complex sentences
Noun–verb ratio	# nouns/# verbs	Number of nouns per verbs. Might reflect specific difficulty with either nouns or verbs
Open-closed ratio	# open class words/# closed class words	Content words versus function words. Open class: content words. Word class accepts new members easily. Closed class: function words. Word class does not easily accept new members. Might reflect specific difficulty with either content or function words
Disfluencies	# of disfluencies/# all words	Difficulties formulating sentences. All forms of disfluencies, including filled pauses and retracing as a percentage of all words
Pause to word ratio	# pauses/# all words	Indication of processing speed. Measures how many pauses are needed to formulate one word

**Table 3:** Language characteristics between groups.

	Schizophrenia patients	Healthy controls	Test statistics	
			*F* value	*p*-value
*N*	26	22		
Language variables, mean (SD)				
Total number of words^a^	2430.0 (955.48)	3106.7 (773.75)	–	–
Articulation rate	4.2 (0.35)	4.5 (0.39)	7.724	0.008**
Pause duration	0.99 (0.246)	0.90 (0.141)	1.580	0.215
Speaking turn duration	8.2 (5.50)	10.6 (6.64)	2.504	0.121
Percentage of time speaking	71.9 (10.09)	78.2 (8.65)	7.051	0.011*
MLU	14.2 (7.55)	18.2 (6.84)	4.803	0.034*
TTR	0.18 (0.035)	0.15 (0.020)	7.369	0.009**
Clauses per utterance	0.57 (0.021)	0.58 (0.015)	11.592	0.001**
Noun–verb ratio	0.71 (0.081)	0.69 (0.074)	0.689	0.411
Open-closed ratio	0.84 (0.073)	0.81 (0.062)	2.804	0.101
Percentage of disfluencies	6.1 (2.22)	5.7 (2.32)	0.152	0.699
Pause to word ratio	12.9 (3.79)	10.9 (3.17)	3.404	0.072

*SD* standard deviation, *N* sample size, *MLU* mean length of utterance, *TTR* type-token ratio.

See Table 2 for additional information on the language variables.

*Indicates significance at the level of *α* = 0.05.

**Indicates significance at the level of *α* = 0.01.

^a^Not included in the MANOVA analyses, only displayed for referential purposes.

A binary logistic regression model was used to investigate to what extent language variables predict group status. The optimal model had high predictive power (Nagelkerke approximation: *R*^2^ = 0.733), and the Hosmer–Lemeshow test for goodness-of-fit was non-significant (*p* = 0.874). This model included mean length of utterance (MLU) and clauses per utterance; age and years of education were entered as covariates. Patients and healthy controls could be classified with this model with a sensitivity of 88.5% and a specificity of 81.8%.

### Symptomatology

We found a significant negative correlation between PANSS negative subscale and articulation rate (*r* = −0.414, *p* = 0.036), speaking turn duration (*r* = −0.420, *p* = 0.033), percentage of time speaking (*r* = −0.715, *p* < 0.001) and MLU (*r* = −0.393, *p* = 0.047). A significant positive association was found between PANSS negative and open-closed ratio (r = 0.397, *p* = 0.044). After false discovery rate (FDR) correction, only percentage of time speaking remained significant (*p* < 0.001*)*. Item-based correlation analyses were performed for PANSS negative items (Supplementary Table 3). PANSS positive and general total subscales revealed no significant associations with the language variables. Exploratory post hoc analyses per PANSS item (Supplementary Table 4), showed correlations between conceptual disorganization and turn duration (*r* = −0.420, *p* = 0.033), percentage of time speaking (*r* = −0.715, *p* < 0.001), MLU (*r* = −0.393, *p* = 0.047), and open-closed ratio (i.e., a ratio of content words versus function words) (*r* = 0.397, *p* = 0.044). Excitement was associated with articulation rate (*r* = 0.501, *p* < 0.001), whereas grandiosity was positively associated with percentage of time speaking (*r* = 0.415, *p* = 0.035).

### White matter integrity

**Fig. 2:**
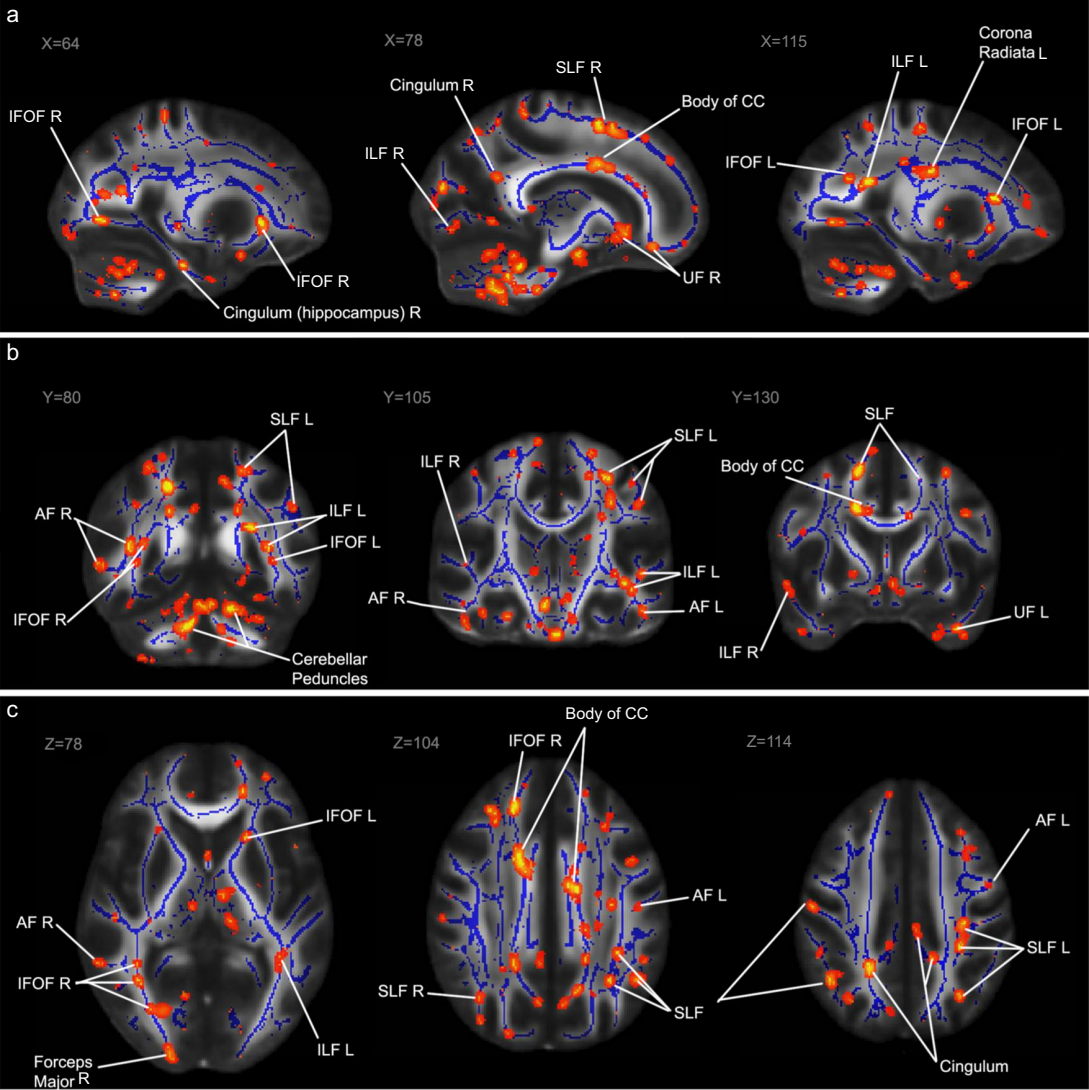
Results of tract-based spatial statistics (TBSS) analysis. The areas highlighted in red/yellow indicate significantly reduced fractional anisotropy FA values for the patient group compared to the control group after correction for multiple comparisons. These results are projected on an FMRIB58 FA standard brain and the mean FA skeleton derived from our sample (*n* = 48), in blue. Some of the regions of interest and other areas with significant differences are labeled in both hemispheres (L: left, R: right), among which the superior longitudinal fasciculus (SLF), inferior longitudinal fasciculus (ILS), inferior fronto-occipital fasciculus (IFOF), arcuate fasciculus (AF), the uncinate fasciculus (UF) and the corpus callosum (CC). Areas with significant differences are labeled in the sagittal plane (**a**), the coronal plane (**b**) and the axial plane (**c**).

Two separate MANCOVA’s were used to determine whether healthy controls and patients with schizophrenia differed on DTI measures of both the (skeletonized) language tracts and the whole brain. The results revealed no overall differences between healthy controls and patients with schizophrenia on mean FA values (*F*_(11,35)_ = 0.783, Pillai’s trace = 0.197, *p* = 0.655) or mean MD values (*F*_(11,33)_ = 1.351, Pillai’s trace = 0.310, *p* = 0.242). However, voxel-wise analyses with Tract-Based Spatial Statistics (TBSS) revealed significantly decreased clusters of voxels in the patients in all ROIs, as well as the corpus callosum, cingulum and the corona radiata (Fig. 2). Our primary analyses concerning FA are presented here; our secondary analyses concerning MD are presented in the supplementary material (Supplementary Tables 5, 6).

Multivariate linear regression analyses revealed that language measures explained 46.7% of the variance of the mean FA of the language tracts and 51.6% of the whole brain mean FA in patients with schizophrenia (Table 4).

**Table 4:** Relation between language disturbances and fractional anisotropy (FA).

Dependent variable	Significant predictor(s)	*B*	Confidence interval *B* (95%)		Beta	Adjusted *R*^2^	Anova	
							*p* (uncorr.)	*p* (FDR corr.)
Healthy controls								
Mean FA language tracts	MLU	0.001	0.0005	0.002	0.557	0.483	0.002**	0.003**
	Clauses per utterance	−0.351	−0.675	−0.028	−0.367			
	Pause to word ratio	−0.002	−0.004	−0.0004	−0.426			
Mean FA whole brain	Speaking turn duration	0.001	0.0003	0.002	0.567	0.331	0.016*	0.016*
	Clauses per utterance	−0.315	−0.616	−0.014	−0.416			
Schizophrenia patients								
Mean FA language tracts	Pause duration	0.031	0.008	0.053	0.416	0.467	0.001**	0.002**
	MLU	−0.001	−0.002	0.000	−0.383			
	Noun–verb ratio	−0.095	−0.175	−0.016	−0.426			
Mean FA whole brain	Pause duration	0.024	0.007	0.042	0.410	0.516	0.001**	0.002**
	Speaking turn duration	0.001	0.0001	0.002	0.394			
	MLU	−0.001	−0.001	−0.00007	−0.363			
	Noun–verb ratio	−0.080	−0.142	−0.019	−0.445			

Significant predictors for the DTI values are displayed. The adjusted *R*^2^ and ANOVA *p*-values display the fit and significance of the full model.

*FDR* false discovery rate, *uncorr.* uncorrected, *corr.* corrected, *MLU* mean length of utterance.

*Indicates significance at the level of *α* = 0.05.

**Indicates significance at the level of *α* = 0.01.

In healthy controls, language variables were also highly explanatory of mean FA of the language tracts (48.3%) and whole brain FA (33.1%). Regression analyses for the ROIs individually are summarized in Table 5.

**Table 5:** Relation between fractional anisotropy (FA) and language disturbances per ROI.

Dependent variable	Significant predictor(s)	*B*	Confidence interval *B* (95%)		Beta	Adjusted *R*^2^	Anova	
							*p* (uncorr.)	*p* (FDR corr.)
Healthy controls								
AF left	Pause duration	0.037	0.004	0.071	0.318	0.753	0.000152**	0.002**
	MLU	0.002	0.001	0.003	0.844			
	Clauses per utterance	−0.606	−0.908	−0.304	−0.556			
	Disfluencies	0.003	0.001	0.005	0.380			
	Pause to word ratio	−0.004	−0.006	−0.002	−0.770			
AF right	MLU	0.001	0.0004	0.002	0.481	0.465	0.002**	0.004**
	Clauses per utterance	−0.556	−0.970	−0.143	−0.463			
	Pause to word ratio	−0.002	−0.004	−0.001	−0.432			
IFOF left	MLU	0.001	0.0004	0.002	0.528	0.418	0.005**	0.007**
	Pause to word ratio	−0.002	−0.004	−0.0003	−0.429			
IFOF right	Speaking turn duration	0.001	0.001	0.002	0.558	0.416	0.005**	0.007**
	Clauses per utterance	−0.468	−0.902	−0.034	−0.401			
	Pause to word ratio	−0.003	−0.005	−0.001	−0.466			
ILF left	Speaking turn duration	0.002	0.0004	0.003	0.766	0.652	0.001**	0.003**
	MLU	0.001	0.0003	0.002	0.501			
	Clauses per utterance	−0.430	−0.763	−0.097	−0.442			
	Disfluencies	0.003	0.0004	0.005	0.461			
	Pause to word ratio	−0.003	−0.005	−0.002	−0.735			
ILF right	Speaking turn duration	0.002	0.001	0.003	0.591	0.329	0.017*	0.019*
SLF left	MLU	0.001	0.001	0.002	0.567	0.527	0.001**	0.003**
	Clauses per utterance	−0.341	−0.671	−0.010	−0.332			
	Pause to word ratio	−0.002	−0.004	−0.001	−0.482			
SLF right	Speaking turn duration	0.001	0.0004	0.002	0.512	0.463	0.002**	0.004**
	Clauses per utterance	−0.617	−0.978	−0.257	−0.610			
	Pause to word ratio	−0.002	−0.004	−0.0003	−0.413			
UF left	MLU	0.001	0.0002	0.002	0.435	0.388	0.008**	0.010*
	Clauses per utterance	−0.342	−0.683	−0.0002	−0.363			
UF right	Speaking turn duration	0.001	0.00003	0.002	0.425	0.250	0.043*	0.043*
Schizophrenia patients								
AF left	Pause duration	0.039	0.010	0.069	0.406	0.478	0.001**	0.003**
	MLU	−0.001	−0.002	−0.0003	−0.433			
	Noun–verb ratio	−0.136	−0.240	−0.033	−0.462			
AF right	Turn duration	0.002	0.0006	0.003	0.401	0.126	0.042	0.047*
	Pause to word ratio	0.004	0.0002	0.007	0.612			
IFOF left	Pause duration	0.030	0.006	0.053	0.393	0.464	0.002**	0.003**
	Speaking turn duration	0.001	0.0001	0.003	0.401			
	MLU	−0.001	−0.002	−0.0003	−0.451			
	Noun–verb ratio	−0.085	−0.166	−0.003	−0.370			
IFOF right	Noun–verb ratio	−0.143	−0.206	−0.080	−0.763	0.509	0.001**	0.003**
	Disfluencies	−0.004	−0.006	−0.001	−0.541			
	Pause to word ratio	0.002	0.001	0.004	0.614			
ILF left	Pause duration	0.029	0.008	0.051	0.410	0.494	0.001**	0.003**
	Speaking turn duration	0.001	0.0002	0.002	0.414			
	MLU	−0.001	−0.002	−0.0002	−0.426			
	Noun–verb ratio	−0.083	−0.158	−0.008	−0.384			
ILF right	Pause duration	0.020	0.0001	0.040	0.317	0.448	0.002**	0.003**
	Speaking turn duration	0.001	0.0001	0.002	0.342			
	MLU	−0.001	−0.002	−0.0002	−0.455			
	Noun–verb ratio	−0.091	−0.160	−0.021	−0.473			
SLF left	Pause duration	0.038	0.010	0.066	0.420	0.479	0.001**	0.003**
	MLU	−0.001	−0.002	−0.0001	−0.374			
	Noun–verb ratio	−0.130	−0.226	−0.034	−0.474			
UF left	Pause duration	0.048	0.019	0.078	0.544	0.364	0.002**	0.003**
	Noun–verb ratio	−0.111	−0.200	−0.022	−0.413			
UF right	Pause duration	0.030	0.006	0.053	0.416	0.466	0.002**	0.003**
	Noun–verb ratio	−0.115	−0.191	−0.040	−0.534			
	Disfluencies	−0.005	−0.008	−0.002	−0.636			
	Pause to word ratio	0.003	0.0005	0.005	0.601			

Significant predictors for the DTI values are displayed. The adjusted *R*^2^ and ANOVA *p*-values display the fit and significance of the full model.

*FDR* false discovery rate, *uncorr.* uncorrected, *corr.* corrected. *N/A* not applicable, *MLU* mean length of utterance, *AF* arcuate fasciculus, *IFOF* inferior fronto-occipital fasciculus, *ILF* inferior longitudinal fasciculus, *SLF* superior longitudinal fasciculus, *UF* uncinate fasciculus.

*Indicates significance at the level of *α* = 0.05.

**Indicates significance at the level of *α* = 0.01. No significant model was found for the right SLF in the schizophrenia patients group, therefore this ROI is not displayed here.

## Discussion

The aim of the current study was to investigate how language characteristics relate to schizophrenia pathology, symptom severity and integrity of the white matter language tracts in patients with schizophrenia and healthy controls. Patients with a schizophrenia spectrum disorder showed quantifiable language disturbances; they spoke less, their articulation rate was slower and they used less complex sentences compared to the matched healthy controls. Language analysis can be a helpful aid in diagnosis. Furthermore, there was a strong relation between these decreased language parameters and negative symptoms, suggesting that language analyses are especially helpful to detect negative symptoms. Also, quantitative properties of spoken language output are strongly related to white matter integrity of the language tracts in both patients and healthy controls.

Our results showed that patients with schizophrenia and healthy controls differed on a broad variety of language measures, including speech tempo and the amount of language produced, as well as measures of complexity. Furthermore, we found that analyzing spontaneous language production can be a powerful diagnostic tool, as it distinguishes between patients with schizophrenia and healthy controls with a sensitivity of 88.5% and a specificity of 81.8%. These sensitivity and specificity indices are in the range of blood-based molecular biomarkers (sensitivity and specificity 90%^27^) and neuroimaging markers using machine learning (accuracy varying between 61.1 and 95%^28^) for schizophrenia. The high sensitivity and specificity we found are remarkable, especially given the simplicity of the model and the small number of predictors (four variables). However, it should be noted that our sample is small and further research, including cross-validation, is necessary to assess the full potential of language variables as a diagnostic biomarker.

We showed that language disturbances are associated with PANSS negative, as well as individual items of the PANSS positive and general subscales. However, most associations were no longer significant after FDR correction. The absence of a correlation with total PANSS positive and general scores could be explained by the fact that all patients were medicated and relatively free of overt psychosis at the time of assessment.

In both patients with schizophrenia and healthy controls, several aspects of language proved highly predictive of structural integrity of the language pathways. While the patients with schizophrenia in our sample had disturbances in language production, the mean integrity of their white matter language tracts was similar to that of the healthy controls. However, our results reveal more fine-grained deviations in the integrity of the white matter tracts, revealing patterns of deviating voxel clusters in all language tracts.

More specifically, in healthy controls, the mean FA of the language tracts was predicted by the MLU, clauses per utterance and the pause to word ratio, while the whole brain FA was predicted by speaking turn duration and clauses per utterance. In patients the FA of the language tracts was predicted by pause duration, MLU and noun–verb ratio. The same pattern was found for the mean FA of the whole brain, only speaking turn duration was found as an additional predictor. These results can be explained by at least two aspects of language production. First, MLU, noun–verb ratio and clauses per utterance are measures of sentence complexity; greater utterance length and more clauses or verbs per utterance indicate more complex sentences. In child language acquisition, sentences become longer and more complex with age^29,30^. In general, white matter integrity increases with age in typically developing children^31^, which is highly correlated with language development^32^. Second, pause duration and pause to word ratio are thought to reflect speaking efficiency and/or processing speed. Previous research has shown that FA has been associated with information processing efficiency^33^. Our results confirm that white matter integrity in the language tracts is associated with increased complexity and speaking efficiency in healthy controls, and extends these findings to patients with schizophrenia.

Importantly, the relation between language variables and the integrity of the white matter tracts appears to be more specific in healthy controls than in patients with schizophrenia. In healthy controls, language is a better predictor for the language tracts than for the whole brain FA (adjusted *R*^2^ = 0.483 and 0.331, respectively). In patients, however, the language measures we used were a better (or at least similar) predictor for the whole brain FA than for the FA of the language tracts (adjusted *R*^2^ = 0.516 and 0.467, respectively). This finding can be interpreted as decreased brain specialization, since previous research has hemispheric specialization is decreased in schizophrenia^34,35^. Alternatively, this finding could reflect more general cognitive disturbances in schizophrenia. Schizophrenia is characterized by broad disturbances in cognition, including decreased processing speed, memory deficits and attention problems^36,37^. These cognitive disturbances may lead to disturbances in language that are nonspecific to language, and therefore show less clear associations with language tracts. We further showed that language measures predict white matter integrity better in some language tracts than in others. In healthy controls, up to 75.3% of the variance of the FA of the left AF is explained by aspects of spontaneous speech, while only 25% of the right UF is explained by language measures. Again, this specificity is less profound in patients with schizophrenia, where 47.8% of the variance the left AF is explained by the language measures.

Previous research revealed significant reductions in white matter integrity in patients with schizophrenia as compared to healthy controls^38,39^. In the current study, we did not find any group differences when looking at average FA/MD over an atlas-based ROI. This might be related to the relatively small sample size, or to the fact that most patients had recent onset psychotic illness, whereas previous research suggests that white matter deterioration in schizophrenia increases with duration of illness^40^. However, we did find significant group differences on clusters of voxels using voxel-wise analyses with TBSS in all language tracts, as well as the corpus callosum, cingulum and the corona radiata. These preliminary results suggest abnormalities at the microstructural level may be a part of a diffuse pattern of brain development in recent onset schizophrenia. The biologic interpretation of these microstructural anomalies remains speculative. Schizophrenia, as a neurodevelopmental disorder, has a subtly abnormal circuit underlying cortical and cerebellar functions such as, motor skills, language, cognition and emotions. Multiple mechanisms are likely to be involved^41^ and per individual, some mechanisms may be more important than others. A very early mechanism may stem from the innate immune system of the brain, especially microglia and complement in shaping the developing brain. Inadequate pruning may result in underdeveloped connections. With myelination to follow much used network connections, fewer well trafficked connections will become well myelinated^42^ In mouse models, decreased white matter integrity has been associated with acute axon and myelin damage^43^. However, a definite pathway for abnormal white matter connectivity in schizophrenia remains elusive.

Previous studies have proposed that FTD severity is related to integrity of white matter language tracts^10,19^. This hypothesis is not supported by our data, as we found language disturbances to be present in the absence of large scale white matter aberrations that were confirmed by previous research^18,44,45^. This difference might be related to the fact that previous studies used FTD rating scales to measure disturbances in language. A disadvantage of using symptom-based severity scores such as FTD rating scales is that these are not scored in healthy controls; therefore, this relation was not previously assessed in healthy controls. FTD rating scales may not be sensitive enough to detect subtle or preclinical deviations in language. Our results indicate that aspects of spontaneous language production are strongly related to white matter integrity in both healthy controls and patients with schizophrenia. Furthermore, we have shown that the white matter integrity of language tracts is not distinctive for patients and healthy controls, while the functional language output is. This is in agreement with research on structural and functional brain abnormalities in schizophrenia, which suggests that structural abnormalities (if observable) are modest, and that it is difficult to distinguish brains of patients from those of healthy controls^46,47^. Instead, schizophrenia is associated with complex alterations in regional patterns of activity in the brain, mostly in task related and resting state activity^46–49^.

Interestingly, in the healthy controls, language variables were more predictive of left-hemisphere language tracts than their right hemisphere counterparts. The white matter language network is generally more lateralized towards the left hemisphere in right-handed subjects^50^, although temporo-frontal networks in the right hemisphere support sentence-level prosody^22,51^. Of note, recent functional MRI studies of speech production suggest that language is not localized to the left hemisphere, instead advocating the importance of the right hemisphere during language production^52,53^. However, these studies involved storytelling tasks; a register of speech that involves a great deal of prosody, emotion, humor, and is not considered neutral spontaneous speech^54,55^. This greater emotional involvement in narrative production may in part explain the large right-hemispheric contribution during these tasks^22,51^. Our finding that several language tracts are more left-lateralized in healthy controls thus adheres to current views on the white matter language network^22,24,50,56^.

In the patients, we found no clear pattern of left-hemisphere specificity for the language variables. There is strong evidence for reduced (functional) lateralization in schizophrenia, which is evidenced by increased mixed-handedness^57^ and diminished language lateralization^58–61^. Schizophrenia patients show a reduction of left-lateralization in several white matter language tracts, including the UF^62–64^ and the IFOF^64^. The right-shift of FA of the UF correlates with negative symptoms^64^. Our results confirm these findings, as the right UF in patients was strongly associated with disfluencies and pauses, more than its left hemisphere counterpart.

This study directly assessed the relation between language disturbances and schizophrenia pathology and symptomatology, as well as the integrity of white matter language tracts in patients with schizophrenia and healthy controls. There are a number of limitations to this study. First, to date, there is no white matter atlas that includes a mask for the AF. This tract is still under investigation and the exact anatomy is still disputed^65,66^. Consequently, we used the temporal branch of the SLF as a mask for the AF. This mask was more of an approximation to the AF, and results concerning the AF should therefore be interpreted with caution. As there is limited research on incorporating these specific language and MRI measures, our results highlight the need for replication in a larger independent sample. Third, participants were relatively stable at time of assessment, which precluded the demonstration of correlations with positive symptoms. Further studies across ages, illness severity and disease durations are needed to understand the trajectory of language disturbances in schizophrenia. Especially replication in a group at high-risk for psychosis would be highly valuable to assess whether both white matter abnormalities and language disturbances proceed the occurrence of a psychotic disorder. Fourth, we had no control group that takes antipsychotics, without the presence of a psychotic disorder. Lastly, we used (parental) years of education as a proxy of intelligence, however, we did not control for differences in IQ between the groups. While there is currently no evidence suggesting that IQ correlates with spontaneous speech markers^67^, the influence of IQ on spontaneous speech remains relatively unknown. Therefore, we cannot fully rule out the influence of differences in intelligence on our results.

In conclusion, quantifiable aspects of language are a sensitive and specific tool in the classification of patients with schizophrenia and healthy controls. Furthermore, these language disturbances are associated with symptom severity, especially with negative symptoms. In both patients with schizophrenia and healthy controls, quantifiable aspects of language are highly predictive of the integrity of white matter tracts associated with language. Our current findings make an important contribution to recent to initiatives such as the Research Domain Criteria project (RDoC) and its aim towards precision psychiatry, which advocates a focus on dimensions of neurobiology and observable behavior rather than symptom-based classification systems such as the DSM and ICD^68,69^. Given that language analyses are non-invasive, quickly performed and low-cost, language analyses are a promising tool in schizophrenia, with both clinical and neurobiological validity.

## Methods

### Subjects

A total of 48 participants, 26 patients with a schizophrenia spectrum disorder and 22 healthy controls, were included between 2015 and 2018 at the University Medical Center Utrecht (UMCU), the Netherlands (trial registration number: NCT01999309). Participants were included if they were (1) aged 18 years or above and (2) a native speaker of Dutch. Patients were included if they met criteria for a DSM-IV diagnosis of: 295.x (schizophrenia, schizophreniform disorder, schizoaffective disorder) or 298.9 (psychotic disorder not otherwise specified). Patients were diagnosed by their treating psychiatrist. A neuropsychologist confirmed the diagnosis using the Comprehensive Assessment of Symptoms and History (CASH) interview^70^. Healthy controls were screened for the absence of previous or current mental illness using the CASH by a neuropsychologist. Healthy controls were excluded if a family history of psychotic symptoms was reported. Additional exclusion criteria were the presence of uncorrected hearing disabilities or speech deficits (such as stutter), contraindications for MRI and left-handedness. The severity of psychotic symptoms was assessed in all patients with the PANSS^26^. This study was approved by the ethical review board of the UMCU. Written informed consent was obtained from all participants. Participants received a small monetary award for participation. Antipsychotic drug dosages were recalculated into chlorpromazine equivalents to evaluate treatment effects in the patients^71^.

### Language data acquisition and processing

To elicit spontaneous spoken language we conducted semi-structured interviews with an average duration of fifteen minutes. Participants were informed that this was part of an analysis regarding “general experiences”; only after completion of the interview they were told that the research also focuses on the way they speak. To prevent variations in language due to the topic that was discussed, a standard set of questions was used. All questions concerned “neutral” general life experiences; topics that could be expected to have markedly different emotional valence for patients and healthy controls were not addressed. For instance, topics such as “quality of life” or “health” were avoided. If for any reason a subject did not want to answer a question, the interviewer would move on to the next question. For a list of the questions, see Supplementary Table 7.

An AKG-C544l head-worn cardioid microphone was used to record the subject’s speech. Speech was digitally recorded onto a Tascam DR40 solid state recording device at a sampling rating of 44,100 kHz with 16-bit quantization. The digitized recordings were analyzed using the Praat software^72^, which is standardly used for acoustic analyses of speech. Speech signals of interviewer and participant were separated by hand onto two different digital audio tracts by J.N.d.B. and A.E.V. Each stretch of speech was coded as belonging either to the participant or the interviewer. When both speakers spoke at the same time, that speech segment was coded as belonging to both speakers. The speech segments were recombined into new audio files per participant, which each thus contained only the time that an individual participant was speaking and pausing. Data files were blinded for diagnosis to prevent bias in separating the speaker. Inter-rater reliability for tier separation was 97.7%. All files were set to an average sound pressure level of 60 dB to avoid differences in the analyses based on speaking volume.

The “Praat Script Syllable Nuclei v2”^73^ was used to automatically obtain speech and articulation rates. The output of this script includes the total number of syllables and the total number of pauses. Pauses were defined as silences longer than 200 ms, as shorter silences in speech are often related to the articulation of particular sounds, notably plosives (e.g., the /p/, which introduces a short silence in the sound wave)^74^. The raw measures were recalculated as a percentage of the duration of the participants’ audio track, since they are strongly dependent on the length of the interview. The participants’ audio file was transcribed according to CHILDES-CHAT guidelines^75^. CLAN software applications EVAL and FLUCALC^76^ were used to extract a comprehensive collection of commonly used measures that reflect linguistic fluency and complexity.

This resulted in the following language measures: articulation rate, average pause duration, speaking turn duration, percentage of time speaking, MLU, TTR, clauses per utterance, noun–verb ratio, open-closed ratio, disfluencies and pause to word ratio; for additional information on these variables, see Table 2.

### DTI acquisition and analysis

MRI scanning was performed by trained MR technicians using a Philips Achieva 3 tesla scanner (Philips Medical Systems, Best, the Netherlands) at the UMCU equipped with an eight-channel SENSE head coil.

Two transverse echo planar imaging diffusion-weighted single shot spin-echo scans were acquired (*b*-value = 1000 s/mm^2^, 30 non-collinear diffusion directions; 5 diffusion-unweighted (*b* = 0 s/mm²) volumes; field of view = 240 mm × 240 mm; acquisition matrix = 128 × 128; reconstruction matrix = 128 × 128; flip angle = 90°; slice thickness = 2 mm, 75 consecutive slices; TE = 68 ms; TR = 7011 ms; parallel imaging factor (SENSE) = 3; no cardiac gating). For the second diffusion-weighted scan the *k*-space readout direction was reversed (anterior–posterior) enabling a correction for susceptibility artefacts in the post processing step.

Participants were allowed to watch television and were requested not to move during the scanning procedures. DTI data was preprocessed using the Diffusion Toolbox implemented in FMRIB Software Library (FSL) release 5.0.9 (ref.^77^). The Brain Extraction Tool was used to remove the skull and other non-brain areas and to create a binary brain mask^78^. The FSL topup tool was used to compute the susceptibility correction parameters using the diffusion-unweighted volumes from both the DTI scans. These parameters where included into FSL’s eddy program enabling simultaneous correction of the DTI data for susceptibility artefacts, head motion and eddy current distortion.

A diffusion tensor model was then fitted to every voxel, and FA and MD maps were created. FA and MD are thought to reflect the coherence of the fiber orientation and free-water concentrations^79,80^. TBSS was used to perform voxel-wise statistical analysis on the FA and MD maps^81^. First, we ran a non-linear registration of all the subjects FA and MD images to the FMRIB58_FA image in MNI152 space. Next, the average FA map of all subjects was skeletonized by eliminating all voxels with FA < 0.2, and this skeleton mask was used to obtain both FA and MD data from all of our subjects. The registered FA and MD maps in standard space (computed as part of the TBSS analysis) were used for a ROI based analysis. Using the FA and MD maps, mean FA and MD values were computed for several predefined ROIs. The following ROIs were included: the SLF, AF, ILF, IFOF and UF bilaterally. The Johns Hopkins University (JHU) ICBM-DTI-81 white matter labels atlas (for bilateral SLF and UF) and the JHU white matter tractography atlas (for bilateral AF, ILF and IFOF) in MNI space were used to create masks from which average FA and MD values were extracted^82,83^.

In addition to exploring group differences based on the atlas masks, the skeletonized FA and MD images were used to conduct voxel-wise statistical comparison using FSL’s randomize function^84^. With this analysis, group differences can be seen at the voxel-level, rather than for the whole ROI. Therefore, subtle differences in clusters of voxels can also be detected.

### Statistical analyses

All statistical analyses were performed using the Statistical Package for Social Sciences (SPSS, version 25). Independent samples *t*-tests were performed to test for group differences on demographic variables. A *χ*^2^ test was performed for categorical variables. First, group differences for the language measures were tested by performing a one-way MANCOVA, controlling for age. Next, we investigated: (1) which language variables were associated with group membership (healthy controls vs patients), by performing a backward binary logistic regression. Predictors were the language variables, as well as age, gender and education level; (2) which language variables were associated with psychotic symptoms, by performing correlation analyses were between PANSS items and language variables; (3) whether severity of language disturbances were associated with white matter aberrations in the ROIs, through backwards multivariate linear regression analyses. Mean FA values from the ROIs as well as whole brain FA and mean FA of the language tracts were entered as dependent variables and language variables as independent variables. This analysis was repeated for the MD values. To account for multiple comparisons, FDR was employed^85^.

### Reporting Summary

Further information on research design is available in the Nature Research Reporting Summary linked to this article.

## Supplementary information

Supplemental material

Reporting summary

Supplementary Tables

## Data Availability

The data that support the findings of this study are available on request from the corresponding author. The data are not publicly available due to them containing information that could compromise research participant privacy or consent.
