# Supplementary material for: Language in schizophrenia: relation with diagnosis, symptomatology and white matter tracts
Source: NPJ Schizophr. 2020 Apr 20;6:10. doi: 10.1038/s41537-020-0099-3 (PMC7171150; doi:10.1038/s41537-020-0099-3)
Supplement: Supplementary file 1 — Supplemental material [file 41537_2020_99_MOESM1_ESM.docx]

**Supplementary material**

**Supplementary Table 1 Questions used in the semi-structured interview**

| 1 | Kun je vertellen over je zwemles van vroeger? Hoe vond je dat? Wat vond je het moeilijkst? En het leukst? |
| --- | --- |
|  | *Can you tell about your swimming lesson from when you were young? How did you like it? What did you find the most difficult? And what did you like the best?* |
| 2 | Kun je vertellen over je tandartservaringen (slechte en goede ervaringen)? Kun je bijvoorbeeld vertellen over de laatste keer dat je bent geweest? En hoe heb je dat als kind ervaren? |
|  | *Can you tell about your dentist experiences (bad and good experiences)? For example, can you tell me about the last time you've been? And how did you experience going as a child?* |
| 3 | Heb je een rijbewijs? Zo ja, kun je wat vertellen over hoe de lessen gingen en hoe het examen ging? |
|  | *Do you have a driving license? If so, can you tell me how the lessons went and how the exam went?* |
| 4 | Ben je wel eens in een pretpark geweest? Kun je daar wat over vertellen? Heb je een favoriet pretpark, vertel daar eens over? Waarom is het je favoriete park? |
|  | *Have you ever been to an amusement park? Can you tell me about that? Do you have a favorite amusement park, tell me about it? Why is it your favorite park?* |
| 5 | Naar welke Nederlandstalige Tv-programma’s kijk je vaak? En aan welke heb je een hekel? Waarom? |
|  | *Which Dutch TV shows do you often watch? And which do you hate? Why?* |
| 6 | Kijk je wel eens naar sport, zoals voetbalwedstrijden? Zo ja, naar welke wedstrijden en wat vind je daarvan? En ben je een fan van een bepaalde club of sporter? |
|  | *Do you ever watch sports, like football matches? If so, what matches and what do you think about it? And are you a fan of a particular club or athlete?* |
| 7 | Kun je je laatste droom beschrijven? |
|  | *Can you describe your last dream?* |
| 8 | Hoe was je laatste verjaardag? Hoe vier je normaal je verjaardag? |
|  | *How was your last birthday? How do you usually celebrate your birthday?* |
| 9 | Wat zou je doen als je een miljoen zou winnen? |
|  | *What would you do if you were to win a million?* |
| 10 | Als je voor altijd een bepaalde leeftijd kon hebben, welke leeftijd zou dat dan zijn? Waarom? |
|  | *If you could have a certain age forever, what age would it be? Why?* |
| 11 | Als je overal ter wereld heen mocht, waar zou je heengaan? |
|  | *If you could go anywhere in the world, where would you go?* |
| 12 | Welk klusje in huis vind je echt vreselijk om te doen? Waarom? Doe je het dan? |
|  | *What kind of household chore do you really hate? Why? Do you do it then?* |
| 13 | Ga je wel eens op vakantie? Wat is je favoriete vakantiebestemming? Waarom? |
|  | *Do you go on vacation? What is your favorite vacation destination? Why?* |
| 14 | Welke oorlog in de geschiedenis heeft indruk op je gemaakt? Waarom? |
|  | *What war in history has impressed you? Why?* |
| 15 | Wat voor werk doen/deden je ouders? |
|  | *What kind of work do your parents / did your parents do?* |
| 16 | Wat voor werk doe je/heb je recent gedaan? Wat sprak je daarin aan? |
|  | *What kind of work do you / have you done recently? What did you talk about?* |
| 17 | Kun je wat vertellen over je laatste sollicitatiegesprek? Was je zenuwachtig? Ben je aangenomen? |
|  | *Can you tell me about your last job interview? Were you nervous? Were you hired?* |
| 18 | Wat is je favoriete jeugdherinnering? Wie waren erbij? Waarom vind je dat zo’n fijne herinnering? |
|  | *What is your favorite childhood memory? Who were there? Why do you think that's a nice memory?* |
| 19 | Hoe werden bij jou thuis de feestdagen gevierd? |
|  | *How did you celebrate the holidays back home?* |
| 20 | Waar ben je geboren? Welke stad, ziekenhuis of thuis? Wie waren daarbij? |
|  | *Where were you born? Which city, in hospital or at home? Who were there?* |
| 21 | Wat voor soort kind was je toen je klein was? |
|  | *What kind of child were you when you were small?* |
| 22 | Had je vroeger een lievelingsknuffel? Of iets anders dat je altijd bij je wilde hebben? |
|  | *Did you have a favorite toy when you were young? Or something else that you always wanted to have with you?* |
| 23 | Wat was je favoriete boek toen je klein was? En waarom was je daar zo dol op? |
|  | *What was your favorite book when you were small? And why were you so fond of it?* |
| 24 | Lijk je op je ouders? Op wie lijk je het meest, waarom? |
|  | *Do you look like your parents? Who do you resemble the most, why?* |
| 25 | Heb je broers of zussen? Op wie lijk je het meest, waarom? Met wie had je het meest ruzie? |
|  | *Do you have any brothers or sisters? Who do you resemble the most, why? Who did you argue with most?* |
| 26 | Wat wilde je vroeger worden (wat voor baan)? Heb je dat lang gedacht? Zou je dat nu nog willen? |
|  | *What did you want to become when you were young (what kind of job)? Did you want that for a long time? Would you still like that?* |
| 27 | Heb je wel eens een huisdier gehad? Zou je een huisdier willen? Wat voor een? Hoe was je band daarmee? |
|  | *Have you ever had a pet? Would you like a pet? What kind? How was your relationship with it?* |
| 28 | Welke mensen waren belangrijk voor je in je jeugd? Waarom? |
|  | *What people were important to you in your youth? Why?* |
| 29 | Was je weleens ziek als kind? Heb je weleens in het ziekenhuis gelegen? |
|  | *Were you sometimes sick as a child? Have you ever been to the hospital?* |
| 30 | Wat was je favoriete eten toen je klein was? |
|  | *What was your favorite food when you were small?* |
| 31 | Wat deed je vroeger waardoor je in de problemen kwam? Werden je ouders dan boos? |
|  | *What did you do in the past that caused you to get into trouble? Did your parents get angry?* |
| 32 | Wat voor spellen speelde je vroeger? Waar speelde je meestal? Buiten/binnen? Met wie? |
|  | *What games did you play when you were young? What did you usually play? Outside inside? With whom?* |
| 33 | Kun je je de laatste keer herinneren dat je goed nieuws kreeg? Wat voor nieuws was dat? Wat voor effect had dat op je? |
|  | *Can you remember the last time that you got good news? What kind of news was that? What effect did that have on you?* |
| 34 | Hoe heb je je beste vriend/in ontmoet? |
|  | *How did you meet your best friend?* |
| 35 | Ben je wel eens verliefd geweest? Weet je nog hoe jullie in gesprek kwamen? Was je verlegen? Weet je nog hoe jullie eerste afspraakje was? Was je zenuwachtig? |
|  | *Have you ever fallen in love? Do you still know how you got talking? Were you shy? Do you remember how your first date was? Were you nervous?* |
| 36 | Wat zijn belangrijke momenten in je leven geweest? |
|  | *Looking back, what were important moments in your life?* |
| 37 | Welke mensen spelen nu een belangrijke rol in je leven? Waarom? |
|  | *Which people now play an important role in your life? Why?* |
| 38 | Wat was een van de beste feestjes waar je ooit bent geweest? |
|  | *What was one of the best parties you've ever been to?* |
| 39 | Als je een tijdmachine had, naar welke tijd in de toekomst/verleden zou je gaan? Waarom? |
|  | *If you had a time machine, what time in the future / past would you go? Why?* |
| 40 | Als je met één persoon op aarde nu mocht spreken, met wie zou dat zijn? |
|  | *If you were to speak to one person on earth, who would that be?* |
| 41 | Als je een dier was, welk dier zou je dan zijn en waarom? |
|  | *If you were an animal, what animal would you be and why?* |
| 42 | Wat is je favoriete bezigheid in de zomer? Waarom? |
|  | *What is your favorite activity in the summer? Why?* |
| 43 | Stel je wordt gedropt op een onbewoond eiland en moet daar een jaar blijven, welke 3 dingen zou je dan meenemen? |
|  | *Imagine being dropped on an uninhabited island and staying there for a year, what 3 things would you bring?* |
| 44 | Als je één van je zintuigen op moest geven welke zou het dan zijn? (horen, zien, voelen, ruiken of proeven) Waarom? |
|  | *If you had to give up one of your senses, what would it be? (hear, see, feel, smell or taste) Why?* |
| 45 | Wat is het leukste cadeau dat je ooit hebt gekregen? Van wie kreeg je het? |
|  | *What is the best gift you ever received? Who did you get from?* |
| 46 | Wat is je favoriete stripfiguur? Waarom die? |
|  | *What is your favorite cartoon character? Why that one?* |
| 47 | Wat is het beste/leukste dat je deze week is overkomen? |
|  | *What is the best / fun thing you have come across this week?* |
| 48 | Wat is het vreemdste dat je ooit hebt gegeten? (bv. slak, oester) |
|  | *What is the strangest thing you have ever eaten? (e.g. snail, oyster)* |
| 49 | Als je vandaag één wereldprobleem mocht oplossen, welk probleem zou dat zijn? Waarom? |
|  | *If you could solve one world problem today, what problem would that be? Why?* |
| 50 | Als je nu een auto mocht kopen, wat voor auto zou dat dan zijn? |
|  | *If you were to buy a car, what kind of car would it be?* |
| 51 | Wat voor soort huis is je droomhuis? Waar zou dit huis staan? Met wie zou je er willen wonen? |
|  | *What kind of house is your dream home? Where would this house be? Who would you like to live with?* |
| 52 | Welke taal zou je nog willen leren spreken? Waarom deze taal? |
|  | *What language would you still like to learn? Why this language?* |
| 53 | Als je voor één dag God zou zijn, wat zou je dan doen? |
|  | *If you could be God for one day, what would you do?* |
| 54 | Hoe ziet jouw perfecte pizza eruit? Wat zit er allemaal op? |
|  | *What does your perfect pizza look like? What's on it?* |
| 55 | Wat is het raarste kledingstuk dat je ooit hebt gedragen? |
|  | *What's the strangest piece of clothing you've ever worn?* |
| 56 | Wat zou je doen als je een dag onzichtbaar zou kunnen zijn? |
|  | *What would you do if you could be invisible one day?* |
| 57 | Van welk beroep droomde je als kind? Wat trok je hier toen aan aan? |
|  | *What profession did you dream of becoming as a child? What did you like about it?* |
| 58 | Als je getuige zou kunnen zijn van elke gebeurtenis in het verleden, heden of toekomst, welke zou het dan zijn? |
|  | *If you could witness any event in the past, present or future, what would it be?* |
| 59 | Als je elke willekeurige fictieve persoon zou kunnen zijn, wie zou je dan kiezen? |
|  | *If you could be any fictional person, who would you choose?* |
| 60 | Als je uit iedereen in de wereld kon kiezen, met wie zou je dan uit eten willen? |
|  | *If you could choose from anyone in the world, who would you like to have dinner with?* |
| 61 | Heb je liever een privévliegtuig of een privé-eiland? En waarom? |
|  | *Would you rather have a private plane or a private island? And why?* |

Note: the original Dutch sentences are presented first, with the English translations in italics below.

**Supplementary Table 2 Association between Positive And Negative Syndrome Scale (PANSS) negative scale and language and speech variables**

|  |  | **Articula-tion rate** | **Pause duration** | **Turn duration** | **% time speaking** | **MLU** | **TTR** | **Clauses per utter.** | **Noun verb ratio** | **Open closed ratio** | **Dis-fluencies** | **Pause to word ratio** |
| --- | --- | --- | --- | --- | --- | --- | --- | --- | --- | --- | --- | --- |
| N1: Blunted affect | r | -.435* | .201 | -.376 | -.561** | -.571** | .496** | -.063 | .356 | .496** | -.030 | .426* |
|  | p | .026 | .325 | .058 | .003 | .002 | .010 | .758 | .074 | .010 | .883 | .030 |
| N2: Emotional withdrawal | r | -.178 | .147 | -.324 | -.568** | -.186 | .147 | .042 | .419* | .164 | -.087 | .170 |
|  | p | .384 | .475 | .106 | .002 | .363 | .474 | .839 | .033 | .425 | .674 | .406 |
| N3: Poor rapport | r | -.253  .213 | -.064  .758 | -.199  .330 | -.535**  .005 | -.140  .495 | .184  .368 | -.033  .874 | .130  .527 | .175  .394 | -.013  .950 | .157  .443 |
|  | p |  |  |  |  |  |  |  |  |  |  |  |
| N4: Pass. social withdraw | r | -.143 | -.147 | -.290 | -.433* | -.229 | .263 | .288 | .166 | .303 | -.352 | -.102 |
|  | p | .487 | .473 | .151 | .027 | .261 | .195 | .153 | .419 | .133 | .078 | .621 |
| N5: Diff. abstract thinking | r | -.086 | -.299 | -.179 | -.210 | -.101 | -.091 | .209 | .083 | .110 | -.374 | -.241 |
|  | p | .674 | .137 | .381 | .303 | .622 | .659 | .306 | .685 | .594 | .060 | .235 |
| N6: Lack of spontaneity | r | -.512** | -.142 | -.342 | -.573** | -.278 | .403* | -.115 | .123 | .289 | .187 | .349 |
|  | p | .007 | .490 | .088 | .002 | .169 | .041 | .577 | .548 | .153 | .360 | .081 |
| N7: Stereotyping thinking | r | .022 | .205 | .098 | .081 | .001 | -.140 | .159 | -.256 | .022 | -.050 | -.087 |
|  | p | .915 | .315 | .634 | .694 | .995 | .496 | .438 | .207 | .916 | .809 | .674 |
| Table legend: r: correlation coefficient. Significant correlations (at the level of α<.05) are bold. MLU: mean length of utterance, TTR: type token ratio, utter.: utterance, N.A.: not applicable. | | | | | | | | | | | | |

**Supplementary Table 3 Association between Positive And Negative Syndrome Scale (PANSS) and language and speech variables**

|  | | **Articulation rate** | **Pause duration** | **Turn duration** | **% time speaking** | **MLU** | **TTR** | **Clauses per utter.** | **Noun verb ratio** | **Open closed ratio** | **Dis-fluencies** | **Pause to word ratio** |
| --- | --- | --- | --- | --- | --- | --- | --- | --- | --- | --- | --- | --- |
| P1: Delusions | r | .131 | -.002 | -.025 | .001 | .058 | -.354 | .085 | -.105 | -.114 | -.077 | -.202 |
|  | p | .522 | .991 | .904 | .997 | .779 | .076 | .679 | .611 | .580 | .710 | .321 |
| P2: Conceptual disorganization | r | .112 | -.080 | .081 | .415* | -.045 | -.269 | .391* | -.443* | -.140 | -.008 | -.196 |
|  | p | .586 | .698 | .695 | .035 | .829 | .183 | .049 | .023 | .494 | .967 | .336 |
| P3: Hallucinatory behavior | r | -.385 | -.148 | -.359 | -.214 | -.227 | .045 | .024 | .362 | -.077 | .207 | .187 |
|  | p | .052 | .470 | .072 | .294 | .264 | .827 | .909 | .069 | .708 | .311 | .361 |
| P4: Ecitement | r | .203 | -.168 | .049 | .089 | .327 | -.310 | .136 | -.205 | -.130 | -.080 | -.270 |
|  | p | .319 | .411 | .813 | .664 | .103 | .123 | .507 | .316 | .526 | .697 | .183 |
| P5: Grandiosity | r | .119 | -.294 | .119 | .237 | .138 | -.330 | .051 | -.358 | .024 | .007 | -.192 |
|  | p | .564 | .145 | .564 | .245 | .502 | .100 | .805 | .073 | .907 | .974 | .347 |
| P6: Suspiciousness | r | .249 | .230 | .029 | -.080 | -.134 | -.073 | .171 | .103 | .156 | -.506** | -.208 |
|  | p | .219 | .258 | .889 | .697 | .513 | .722 | .405 | .618 | .447 | .008 | .308 |
| P7: Hostility | r | N.A. | N.A. | N.A. | N.A. | N.A. | N.A. | N.A. | N.A. | N.A. | N.A. | N.A. |
|  | p |  |  |  |  |  |  |  |  |  |  |  |
| G1: Somatic concern | r | .331 | .225 | .035 | -.055 | .198 | -.147 | .102 | -.204 | -.075 | -.304 | -.346 |
|  | p | .099 | .270 | .865 | .790 | .333 | .473 | .619 | .317 | .716 | .131 | .084 |
| G2: Anxiety | r | -.133 | .076 | -.325 | -.112 | -.254 | .350 | -.037 | .187 | .333 | .112 | .184 |
|  | p | .516 | .714 | .105 | .586 | .210 | .079 | .856 | .361 | .097 | .585 | .367 |
| G3: Guilt | r | -.080 | .011 | .220 | -.036 | -.065 | -.183 | .265 | .118 | -.079 | -.419* | -.231 |
|  | p | .699 | .959 | .280 | .862 | .753 | .371 | .191 | .564 | .702 | .033 | .256 |
| G4: Tension | r | .075 | .223 | -.082 | -.067 | -.117 | .452* | .057 | .040 | .299 | -.118 | .228 |
|  | p | .715 | .274 | .692 | .744 | .570 | .020 | .783 | .846 | .138 | .566 | .262 |
| G5: Mannerisms | r | -.075 | -.193 | -.203 | -.363 | -.098 | .158 | -.285 | .255 | -.015 | .273 | .370 |
|  | p | .714 | .344 | .319 | .069 | .635 | .442 | .159 | .208 | .943 | .177 | .063 |
| G6: Depression | r | -.380 | .208 | -.414* | -.341 | -.440* | .392* | .083 | .334 | .288 | -.014 | .238 |
|  | p | .056 | .308 | .035 | .088 | .024 | .048 | .688 | .096 | .154 | .945 | .241 |
| G7: Motor retardation | r | -.510** | .060 | -.182 | -.206 | -.388* | .035 | .134 | .034 | .037 | .127 | .363 |
|  | p | .008 | .771 | .374 | .313 | .050 | .864 | .514 | .868 | .858 | .535 | .069 |
| G8: Uncooperativeness | r | N.A. | N.A. | N.A. | N.A. | N.A. | N.A. | N.A. | N.A. | N.A. | N.A. | N.A. |
|  | p |  |  |  |  |  |  |  |  |  |  |  |
| G9: Unusual content | r | .267 | -.034 | .052 | .078 | .066 | -.380 | .276 | -.230 | -.221 | -.259 | -.242 |
|  | p | .187 | .870 | .801 | .704 | .750 | .055 | .172 | .258 | .278 | .201 | .233 |
| G10: Disorientation | r | -.216 | .175 | -.199 | -.119 | -.211 | .054 | -.136 | .250 | -.198 | .431* | .494* |
|  | p | .289 | .392 | .331 | .563 | .301 | .794 | .508 | .219 | .333 | .028 | .010 |
| G11: Poor attention^1^ | r | -.267 | -.088 | -.257 | -.140 | -.131 | .216 | .138 | -.054 | .136 | .287 | .207 |
|  | p | .187 | .670 | .206 | .496 | .524 | .289 | .502 | .792 | .508 | .156 | .311 |
| G12: Lack of insight | r | .259 | -.069 | -.013 | .151 | .036 | -.397* | .363 | -.227 | -.171 | -.120 | -.135 |
|  | p | .201 | .738 | .951 | .462 | .863 | .045 | .068 | .264 | .405 | .559 | .510 |
| G13: Disturb. of volition | r | -.018 | .213 | .037 | .064 | -.186 | -.108 | .381 | -.244 | -.083 | -.153 | -.020 |
|  | p | .932 | .295 | .858 | .754 | .363 | .599 | .055 | .229 | .688 | .457 | .922 |
| G14: Poor impulse control | r | -.253 | -.112 | -.009 | -.032 | -.199 | -.084 | .315 | -.020 | .034 | -.108 | .091 |
|  | p | .213 | .585 | .967 | .876 | .330 | .682 | .117 | .924 | .869 | .599 | .659 |
| G15: Preoccupation | r | .040 | .004 | .149 | .108 | -.162 | -.196 | .149 | -.362 | .229 | .018 | .007 |
|  | p | .845 | .984 | .469 | .599 | .428 | .337 | .467 | .069 | .260 | .930 | .973 |
| G16: Act. social avoidance | r | .011 | -.063 | -.075 | -.131 | -.105 | .016 | .140 | .074 | .140 | -.158 | .051 |
|  | p | .956 | .759 | .714 | .523 | .609 | .938 | .494 | .719 | .495 | .440 | .806 |

Table legend: r: correlation coefficient, N.A.: not applicable, MLU: mean length of utterance, TTR: type token ratio, utter.: utterance. ^1^: no correlation coefficient could be calculated since these variables were absent in the patients.

**Supplementary Table 4 Relation between language and speech disturbances and mean diffusivity (MD)**

| Dependent variable | Significant predictor(s) | | beta | Adjusted R^2^ | Anova |
| --- | --- | --- | --- | --- | --- |
|  | | |  |  | *p*(uncorr.) |
| Healthy controls | | |  |  |  |
| Mean MD language tracts | | MLU | -.513 | .390 | .004** |
|  | | TTR | .421 |  |  |
|  | |  |  |  |  |
| Mean MD whole brain | | MLU | -.545 | .413 | .002* |
|  | | TTR | .405 |  |  |
| Schizophrenia patients | | |  |  |  |
| Mean MD language tracts | | Speaking turn duration | -.442 | .200 | .048* |
|  | | Clauses per utterance | -.361 |  |  |
|  | | Pause to word ratio | -.416 |  |  |
|  | |  |  |  |  |
| Mean MD whole brain | | Speaking turn duration | -.472 | .201 | .053** |
|  | | Clauses per utterance | -.399 |  |  |
|  | | Pause to word ratio | -.430 |  |  |
| Legend: Significant predictors for the DTI values are displayed. The adjusted R^2^ and ANOVA p-values display the fit and significance of the full model. uncorr.: uncorrected, *: indicates significance at the level of α=.05, **: indicates significance at the level of α=.01 | | | | | |

**Supplementary Table 5 Relation between language and speech disturbances and mean diffusivity (MD) per ROI**

| Dependent variable | Significant predictor(s) | beta | Adjusted R^2^ | *Anova* |  |
| --- | --- | --- | --- | --- | --- |
|  |  |  |  | *p* (uncorr.) | |
| Healthy controls |  |  |  |  | |
| AF left | MLU | -.442 | .375  u | .004* | |
|  | TTR | .481 |  |  | |
|  |  |  |  |  | |
| AF right | MLU | -.570 | .570 | .001** | |
|  | Clauses per utterance | .328 |  |  | |
|  | Noun verb ratio | .375 |  |  | |
|  | Pause to word ratio | .331 |  |  | |
|  |  |  |  |  | |
| IFOF left | MLU | -.366 | .269 | .020* | |
|  | TTR | .445 |  |  | |
|  |  |  |  |  | |
| IFOF right | MLU | -.357 | .447 | .006** | |
|  | Clauses per utterance | .355 |  |  | |
|  | Noun verb ratio | .435 |  |  | |
|  | Pause to word ratio | .475 |  |  | |
|  |  |  |  |  | |
| ILF left | Speaking turn duration | -.381 | .262 | .037** | |
|  | TTR | .487 |  |  | |
|  | Clauses per utterance | .418 |  |  | |
|  |  |  |  |  | |
| ILF right | Speaking turn duration | -.587 | .446 | .003** | |
|  | TTR | .484 |  |  | |
|  | Clauses per utterance | .443 |  |  | |
|  |  |  |  |  | |
| SLF left | MLU | -.455 | .422 | .002** | |
|  | TTR | .512 |  |  | |
|  |  |  |  |  | |
| SLF right | MLU | -.646 | .388 | .001** | |
|  |  |  |  |  | |
| UF left | MLU | -.559 | .390 | .004** | |
|  | TTR | .411 |  |  | |
|  |  |  |  |  | |
|  |  |  |  |  | |
| UF right | MLU | -.469 | .181 | .028* | |
| Schizophrenia patients |  |  |  |  | |
| AF left | Speaking turn duration | -.436 | .251 | .025* | |
|  | Clauses per utterance | -.439 |  |  |  |
|  | Disfluencies | -.483 |  |  |  |
|  |  |  |  |  | |
| IFOF left | Speaking turn duration | -.409 | .187 | .057 | |
|  | Clauses per utterance | -.383 |  |  |  |
|  | Disfluencies | -.448 |  |  |  |
|  |  |  |  |  | |
| ILF left | Pause duration | -.288 | .394 | .005** | |
|  | Speaking turn duration | -.487 |  |  |  |
|  | Clauses per utterance | -.511 |  |  |  |
|  | Disfluencies | -.417 |  |  | |
|  |  |  |  |  | |
| ILF right | Speaking turn duration | -.379 | .157 | .082 | |
|  | Clauses per utterance | -.389 |  |  |  |
|  | Disfluencies | -.403 |  |  |  |
|  |  |  |  |  | |
| SLF left | Speaking turn duration | -.474 | .281 | .016* | |
|  | Clauses per utterance | -.432 |  |  |  |
|  | Disfluencies | -.491 |  |  |  |
|  |  |  |  |  | |
| UF left | Noun verb ratio | .524 | .205 | .027* | |
|  | Pause to word ratio | -.422 |  |  |  |
|  |  |  |  |  | |
| UF right | Percentage time speaking | -.394 | .120 | .047* | |

Legend: Significant predictors for the DTI values are displayed. uncorr.: uncorrected, *: indicates significance at the level of α=.05, AF: arcuate fasciculus, IFOF: inferior fronto-occipital fasciculus, ILF: inferior longitudinal fasciculus, SLF: superior longitudinal fasciculus, UF: uncinate fasciculus, MLU: mean length of utterance, TTR: type-token ratio, **: indicates significance at the level of α=.01. No significant models were found for the AF right, IFOF right and SLF left for the patients. These ROIs are therefore not displayed here.
